# Analysis on Bacterial Community of *Noctiluca scintillans* Algal Blooms Near Pingtan Island, China

**DOI:** 10.3390/biology14010101

**Published:** 2025-01-20

**Authors:** Yunguang Liu, Yutong Zhang, Haiyan Yao, Zewen Zheng, Wenbo Zhao, Gang Lin

**Affiliations:** Fujian Key Laboratory of Special Marine Bio-Resources Sustainable Utilization, College of Life Sciences, Fujian Normal University, Fuzhou 350117, China; 15060110810@163.com (Y.L.); zytgzyx000@163.com (Y.Z.); daphneyanng@163.com (H.Y.); zhengzewen2019@163.com (Z.Z.); zwb2632617990@163.com (W.Z.)

**Keywords:** Pingtan Island, *Noctiluca scintillans*, bacterial community structure, *Vibrio*

## Abstract

*Noctiluca scintillans*, known as a global red tide species, is a common red tide species found in Pingtan Island. To examine the bacterial community structure in different environments during the red tide period of *N. scintillans* on Pingtan Island, samples were collected from the Algal Bloom Area (ABA), Transition Area (TA), and Non-Algal Bloom Area (NBA) on 6 April 2022, and the environmental physicochemical factors and bacterial community were determined. The outbreak of *N. scintillans* red tide significantly impacted the water quality and bacterial community structure in the affected sea area. The water quality in the ABA has deteriorated markedly, with the contents of COD, NH_4_^+^-N, and PO_4_^3−^ in the ABA being significantly higher than those in the TA and NBA, while the pH is significantly lower than that in the TA and NBA. The richness, diversity, and evenness of the bacterial community in the ABA are all lower than those of the TA and NBA. For instance, the Shannon index values of the three sampling points are 4.41, 5.41, and 6.37, respectively. At the phylum level, the dominant bacterial phyla in the ABA are Proteobacteria, Firmicutes, and Cyanobacteria; in the TA, they are Proteobacteria, Bacteroidetes, and Firmicutes; and in the NBA, they are Proteobacteria, Bacteroidetes, and Cyanobacteria. At the genus level, the dominant bacterial genera in the ABA are *Vibrio*, *Carnobacterium*, *Candidatus_Megaira*, *Planktomarina*, and *Pseudoalteromonas*; in the TA, they are *Vibrio*, *Planktomarina*, *Lentibacter*, *Glaciecola*, and *Jannaschia*; and in the NBA, they are *Planktomarina*, *Amylibacter*, *NS5_marine_group*, *Aurantivirga*, and *marine_metagenome*. In the ABA, the combined proportion of *Vibrio* and *Carnobacterium* exceeds 50%, with *Vibrio_splendidus* accounting for 93% of the total *Vibrio* population. These research results can provide a scientific basis for clarifying the environmental characteristics and bacterial composition during the large-scale *N. scintillans* red tide in Pingtan Island.

## Simple Summary

*Noctiluca scintillans* is a common red tide species on Pingtan Island that blooms in spring. Samples were collected from the Algal Bloom Area (ABA), Transition Area (TA), and Non-Algal Bloom Area (NBA) at Changjiang Ao sea area, Pingtan Island, to study the bacterial community structure in seawater during *N. scintillans* red tide outbreaks. Both environmental factors and bacterial communities were analyzed. The results indicate that during the outbreak of *N. scintillans* in Pingtan Island, the aquatic environment deteriorated, and the structure of bacterial colonies changed. At the phylum level, the most dominant bacterial phylum in the ABA, TA, and NBA was Proteobacteria. The second-most dominant bacterial phylum differed among the areas; in the ABA, it was Firmicutes, while that in the TA and NBA was Bacteroidota. At the genus level, *Vibrio* was the most dominant bacterial genus in the ABA and TA, while *Planktomarina* was the most dominant genus in the NBA. The second-most dominant genus was *Carnobacterium* in the ABA, *Planktomarina* in the TA, and *Amylibacter* in the NBA. In addition, in the ABA, the combined proportion of *Vibrio* and *Carnobacterium* exceeded 50%. Moreover, during *N. scintillans* blooms, the diversity of bacterial communities in the ABA was significantly lower than that in the TA and NBA. These research findings can clarify the environmental characteristics and bacterial composition during the *N. scintillans* red tide in Pingtan Island.

## 1. Introduction

The rapid proliferation or aggregation of certain microscopic plankton in the ocean under specific physicochemical conditions and nutritional circumstances leads to the discoloration of seawater and causes ecological abnormalities that undermine the structure and function of the ecosystem. Such outbreaks are collectively referred to as red tides or Harmful Algal Blooms (HABs) [1]. These transient and frequently recurring HABs are symptoms of eutrophication, exerting significant stress on ecosystem functions; they often damage ecosystem health directly (e.g., by producing toxins) or indirectly (e.g., through oxygen depletion, alterations in food web structure, and reduced light penetration) [2,3,4,5].

*Noctiluca scintillans*, a large dinoflagellate with a diameter of 400–1000 µm, is a major algal species that can cause red tides and is widely distributed across global marine environments. This species exhibits two forms: green and red. The green form, which contains photosynthetic symbiotic algae, is a mixotrophic organism predominantly found in tropical waters with temperatures ranging from 25 °C to 30 °C, such as the coastal waters of the Indian Ocean and the Tropical Pacific; the red form, lacking a photosynthetic system, is a strictly heterotrophic dinoflagellate and is widely distributed in temperate, subtropical, and tropical coastal waters with temperatures ranging from 10 °C to 25 °C. Consequently, the red form is the predominant bloom-forming species in China’s coastal waters [6,7]. The first report of the *N. scintillans* red tide in China was off the coast of Zhejiang Province in 1933 [8], and since then there have been reports of this red tide in the Bohai Sea, Yellow Sea, East China Sea, and South China Sea [7]. The Pingtan coastline is the sea area where *N. scintillans* red tide occurs most frequently [9]. From 2001 to 2019, *N. scintillans* algal blooms accounted for 58.8% of the total number of algal blooms in the Pingtan Sea area [10]. Although *N. scintillans* is non-toxic, its proliferation can cause water hypoxia and release high concentrations of ammonia into the surrounding environment, resulting in the death of a large number of fish and invertebrates [11]. At the same time, it can prey on the eggs and larvae of fish and copepods as well as small phytoplankton in the food web, which may cause ecosystem imbalance [12].

In marine environments, the complex interplay between algae and bacteria involves planktonic bacteria that can either promote or inhibit algal growth and development [13]. Algal blooms significantly impact bacterial community diversity, composition, and function, thereby altering elemental cycles and microbial food webs in coastal waters [5,14,15]. Research has found that the proliferation of certain “harmful bacteria” associated with algal blooms leads to significant environmental issues and poses potential threats to human and marine animal health [16,17,18]. Studies also suggest that *N. scintillans* may act as a vector for pathogens [19,20] such as *Vibrio cholerae*.

In the Pingtan Sea area, there is extensive utilization of floating raft culture for abalone and cage culture for fish. The occurrence of red tides caused by *N. scintillans* algae significantly impacts these aquaculture practices. Therefore, in this study, we collected seawater from the Algal Bloom Area (ABA), Transition Area (TA), and Non-Algal Bloom Area (NBA) around Pingtan Island during the *N. scintillans* red tide period and obtained the physicochemical indexes and bacterioplankton community data from the three different sea areas for comparison to investigate the ecological effects of the red tide of *N. scintillans*. The results of this study can be used to determine the environmental characteristics and bacterial community composition during the outbreak of *N. scintillans* red tide in Pingtan Island and provide a scientific basis for marine ecological protection.

## 2. Materials and Methods

### 2.1. Survey Time and Position

**Figure 1:**
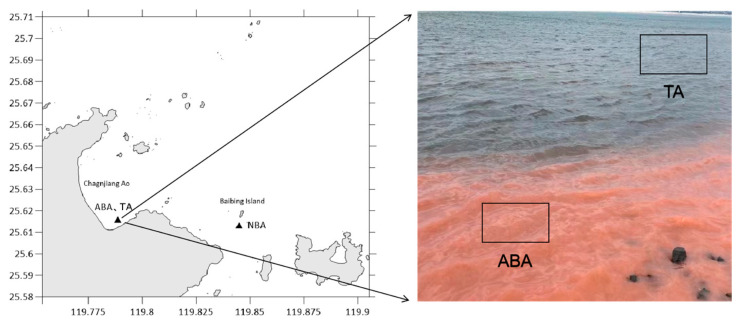
Map of sampling stations located in the Changjiang Ao area of Pingtan Island on 26 April 2022. Abbreviations for the sampling sites are as follows: ABA: Algal Bloom Area, TA: Transition Area, and NBA: Non-Algal Bloom Area.

On 26 April 2022, a *N. scintillans* red tide was identified in the Changjiang Ao area (25.608° N, 119.791° E) of Pingtan Island. The samples were collected from the Algal Bloom Area (ABA), Transition Area (TA) near Changjiang Ao, and Non-Algal Bloom Area (NBA) near Baibing Island (Figure 1).

### 2.2. Sample Collection

A total of 1 L of surface seawater was taken from each of the three sampling areas (ABA, TA, and NBA) and put into sterilized polyethylene bottles. Replicate samples (n = 3) were collected at each site. After filtration with a filter membrane with a pore size of 0.22 μm, the filter paper was put into a 5 mL sterilized centrifuge tube and stored in an ultra-low temperature refrigerator at −80 °C for high-throughput sequencing. The filtrate was stored in the refrigerator at −20 °C to determine physical and chemical indexes.

### 2.3. Determination of Physical and Chemical Indexes

In this study, the physicochemical indexes of seawater were measured, including temperature, salinity, PH, Chemical Oxygen Demand (COD), ammonia nitrogen (NH_4_^+^-N), soluble phosphate (PO_4_^3−^), and Suspended Substance (SS). The water quality index was determined in accordance with The Specification for Marine Monitoring Part 4: Seawater Analysis (GB17378.4-2007) [21].

### 2.4. DNA Extraction and Illumina MiSeq Sequencing

DNA was extracted by using a DNA Kit (Omega Bio-Tek, Norcross, GA, USA). After DNA extraction, the purity of seawater bacteria was determined via 1% agarose gel electrophoresis. Using diluted genomic DNA as a template, the V3~V4 variable region of bacterial 16SrRNA was amplified by using the 338F/806R specific primer barcode. The primer sequences were 338R (5′-ACTCCTACGGGAGGCAGCAG-3′) and 806R (5′-GGACTACHVGGGTWTCTAAT-3′). The PCR amplification system was 25 μL, and the reaction condition was denaturated beforehand at 94 °C for 5 min. Denaturation was carried out at 94 °C for 30 s followed by annealing at 50 °C for 30 s, which was extended at 72 °C for 60 s, 30 cycles, and at 72 °C for 7 min. The reaction was then cooled to 4 °C. After amplification, the PCR amplification products were detected with 1% agarose gel electrophoresis and purified using an Agencourt AMPure XP nucleic acid purification kit. Sequencing libraries were generated using an Illumina TruSeq DNA PCR-Free Library Preparation Kit (Illumina, San Diego, CA, USA), and index codes were added. Finally, the qualified libraries were sequenced on an Illumina MiSeq platform belonging to Beijing Allwegene Tech (Beijing, China).

### 2.5. Data Analysis

The raw data were processed using QIIME (version 1.8.0) software to filter, assemble, and remove chimeras. Sequences with scores below 20, ambiguous bases, primer mismatches, or sequencing lengths shorter than 150 base pairs were eliminated. Effective sequences post-treatment were clustered into operational taxonomic units (OTUs) with 97% consensus using Uparse clustering method, and the species classification information for each OTU was obtained through a comparison with the Silva database.

The alpha-diversity indexes, namely, the Chao1 index, Shannon–Weaver index, and Simpson index, were calculated using Qiime (version 1.8.0) software. A one-way ANOVA was performed using SPSS 20.0 to analyze the significant differences in water quality indexes and diversity of bacterial communities in different samples. The images were generated using Surfer 23, Microsoft Excel, OriginPro 2021 software, and the ChiPlot platform.

## 3. Results

### 3.1. Environmental, Physical, and Chemical Indicators

In this study, temperature, salinity, and pH were relatively similar in the three sampling areas. The PO_4_^3−^, COD, NH_4_^+^-N, and SS values were in the order of ABA > TA > NBA (*p* < 0.05) (Table 1). The levels of PO_4_^3−^ and COD of the ABA and TA exceeded the normal seawater quality standards. It can be seen that all the seawater in the ABA and the TA is of poor class 4.

### 3.2. Diversity Analysis of Bacterial Communities

In this study, we analyzed samples of the ABA, TA, and NBA using 16sRNA high-throughput sequencing technology. A total of 448,749 high-quality sequences and 5065 optimized OTUs were obtained from the three sampling areas. There were some differences in the number of OTUs among the samples from the three sampling areas. The mean number of OTUs in the ABA was significantly lower than that in the other two sea areas (*p* < 0.05). Seawater samples have 99% coverage of the community of all sample bacteria.

Based on the 97% sequence similarity, bacterial OTU sequences selected from the results were analyzed. The results show that the order of the Chao1 index is ABA < NBA < TA. The results of the Shannon index and Simpson index are in the order of ABA < NBA < TA. The diversity indexes of the three sample collection points were significantly different (*p* < 0.05) (Table 2).

### 3.3. Similarity Analysis of Bacterial Communities

The three sampling points had an average of 805 operational taxonomic units (OTUs). Among the points, the NBA had the highest OTU count at 879, followed by the TA with 804, while the TA had the lowest at 731. To understand the co-ownership of bacteria among the samples, a Venn diagram was constructed. The Venn diagram revealed 62 ABA-specific, 84 TA-specific, and 253 NBA-specific OTUs. The three samples shared 429 OTUs, which constituted 53.29% of the total OTUs in each sample, indicating a similar bacterial composition. Notably, the ABA and TA shared the highest number of OTUs (596), suggesting a high similarity between their bacterial communities.

**Figure 2:**
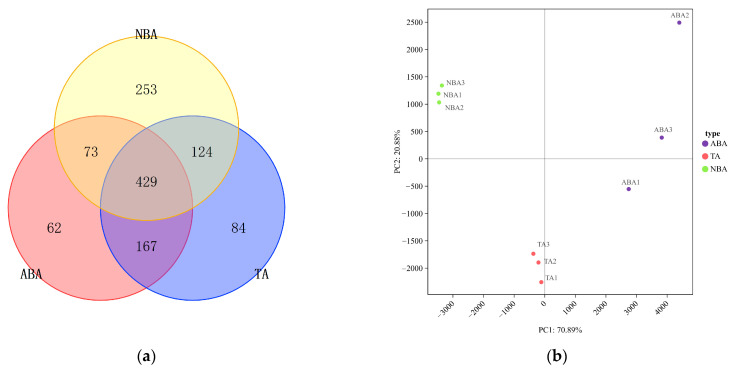
(**a**) Venn diagram of OTU distribution of bacteria in different sampling areas; (**b**) Principal Component Analysis of bacterial community composition in different sampling areas. Abbreviations for the sampling sites are as follows: ABA: Algal Bloom Area, TA: Transition Area, and NBA: Non-Algal Bloom Area.

The PCA analysis results reveal that there is no intersection among the three sampling areas, suggesting significant differences between the groups. The difference between the TA group and NBA group was the slightest, while the difference in the ABA group was greater (Figure 2).

### 3.4. Analysis of Composition of Algal Bacterial Community

**Figure 3:**
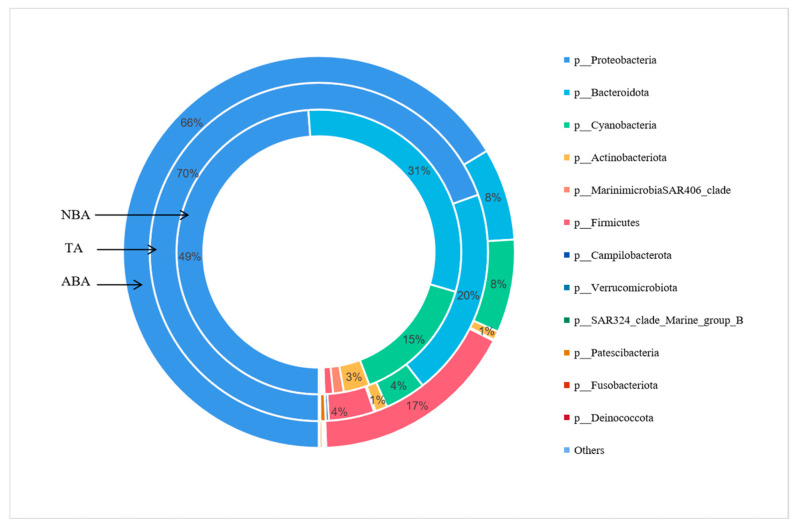
Abundance map of bacterial community at phylum level in different sampling areas. Abbreviations for the sampling sites are as follows: ABA: Algal Bloom Area, TA: Transition Area, and NBA: Non-Algal Bloom Area.

After species annotation, a total of 28 phyla were detected in the three sampling areas: 19 phyla in the ABA, 23 phyla in the TA, and 26 phyla in the NBA. Figure 3 shows the top ten phyla in terms of relative abundance, while the rest are combined into Others. The main bacterial communities in the three sampling areas were Proteobacteria, Bacteroidota, Cyanobacteria, Actinobacteriota, MarinimicrobiaSAR406_clade, Firmicutes, Campilobacterota, Verrucomicrobiota, SAR324_clade_Marine_group_B, Patescibacteria, Fusobacteriota, and Deinococcota. Proteobacteria were the most dominant bacterial community in the three sampling areas, and the relative abundances of the ABA, TA, and NBA were 66.38%, 69.52%, and 48.82%. Bacteroides was the second-most dominant bacterial community in the TA and NBA, with relative abundances of 19.97% and 30.70%. The relative abundances of Cyanobacteria were 7.68%, 3.93%, and 14.72%, and Firmicutes was the subdominant bacterial community in the ABA, with relative abundances of 16.91%, 4.36%, and 0.97%.

**Figure 4:**
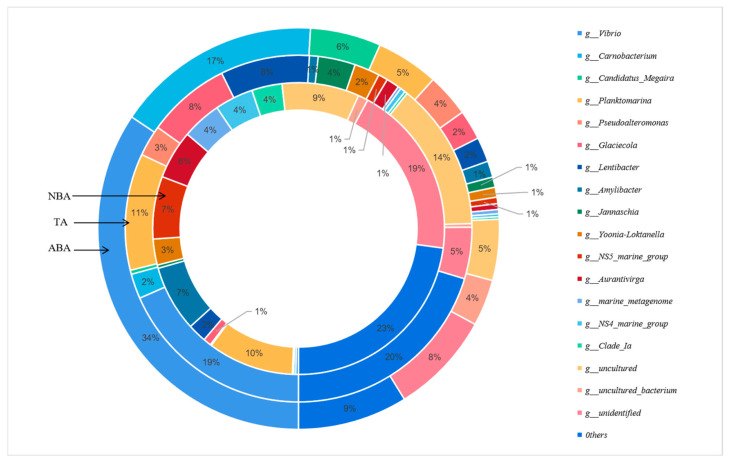
Abundance map of bacterial community at genus level in different sampling areas. Abbreviations for the sampling sites are as follows: ABA: Algal Bloom Area, TA: Transition Area, and NBA: Non-Algal Bloom Area.

At the genus level, a total of 380 bacterial genera were detected in the three sampling areas, of which 267 genera were detected in the ABA, 289 genera in the TA, and 288 genera in the NBA. The main bacterial communities in the three regions were *Vibrio*, *Carnobacterium*, *Candidatus_Megaira*, *Planktomarina*, *Pseudoalteromonas*, *Glaciecola*, *Lentibacter*, *Amylibacter*, *Jannaschia*, *Yonia-Loktanella*, *NS5_marine_group*, and *Aurantivirga* (Figure 4). The species and content of the ABA, TA, and NBA were different at the genus level. *Vibrio* was the most dominant genus in the ABA and TA, with relative abundance of 34.38% and 18.43%, respectively, and a relatively low proportion of 0.31% in the NBA. *Carnobacterium* was the second-most dominant genus in the ABA, with a relative abundance of 16.57%, which is higher than that of the other two regions (*p* < 0.05). The content of *Candidatus_Megaira* in the ABA was 5.69%, which was significantly higher than that in the other two areas (*p* < 0.05). The genus *Planktomarina* fertilized in the ABA with a concentration of 5.04%, lower than that of the TA and the NBA, and it was the most dominant genus in the NBA. The content of unidentified bacteria in algal blooms was in the order of ABA < TA < NBA.

**Figure 5:**
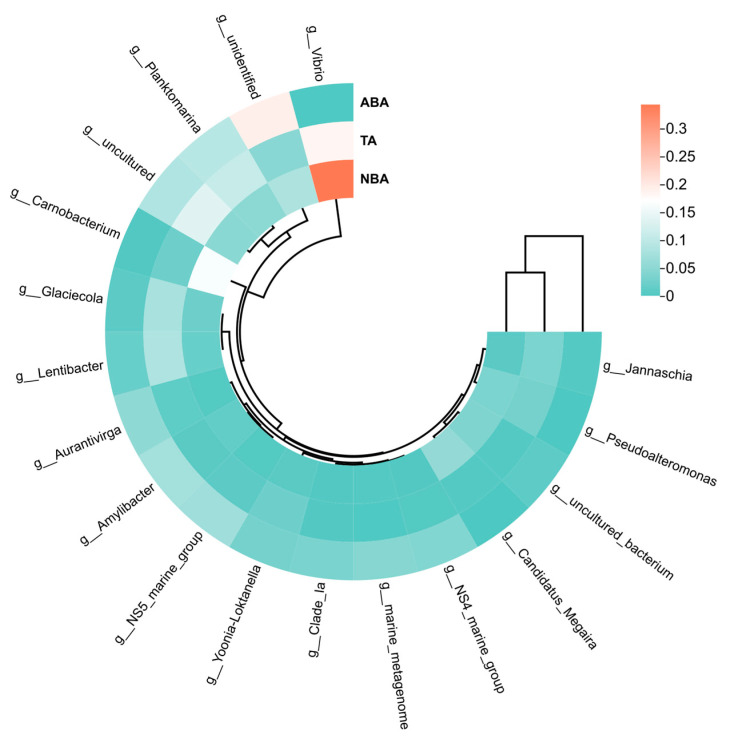
Clustering heat maps of dominant genera of bacterial communities at different sampling areas. Abbreviations for the sampling sites are as follows: ABA: Algal Bloom Area, TA: Transition Area, and NBA: Non-Algal Bloom Area.

The cluster analysis of the dominant genera of different algal bacterial communities showed that the ABA and TA cluster into one branch, suggesting that there was a negligible difference in the structure of bacterial communities among the three marine areas. *Vibrio* was the most dominant bacterium in the ABA and is distinguished from the other two algal bacterial genera, followed by *Carnobacterium*. The dominant bacterium genus of the TA was still *Vibrio*, followed by *Panktomarina*. The proportion of unidentified bacterial genera in the NBA was higher than that in the other two sea areas (Figure 5).

In order to understand the dominant bacterial genera in each sample, the microbial species with relative abundance greater than 1% were identified as the dominant species (Table 3). The dominant species and quantity of bacteria were different in different algal environments. Including unclassified or uncultured bacterial genera, the number of dominant bacterial genera in each sample was 26: 11 in the ABA, 15 in the TA, and 16 in the NBA. Some strains were dominant only in one sea area, among which *Candidatus_Megaira* was dominant in the ABA. *NS3a_marine_group*, *Jannaschia, Litoricola*, *Nautella*, and *Marinomonas* were the dominant strains of the TA. *NS5_marine_group*, *marine_metagenome*, *NS4_marine_group*, *Clade_Iag__Candidatus_Actinomarina*, *OM60_NOR5_clade*, *unidentified_marine_bacterioplankton*, and *Salinisphaera* were the dominant species in the NBA. Several species, such as unidentified, *Planktomarina*, uncultured, and *Lentibacter*, were the dominant species in all three sampling areas.

A total of 15 *Vibrio* species were detected in this study. Among them, *Vibrio_splendidus*, *Vibrio_harveyi_NBRC_15634_ATCC_14126*, *uncultured_vibrio_vibrio_vibrio_Cyclitrophicus_FF75*, *Vibrio_halioticoli_NBRC_102217*, *Vibrio_sp._INTCD148H*, *Vibrio_sp._H075*, and *Vibrio_cholerae* were the main *Vibrio* species (Table 4). In addition, *Vibrio_splendidus* was the most dominant species, accounting for 93% of the total number of *Vibrio*.

### 3.5. Redundancy Analysis of Dominance and Environmental Factors

**Figure 6:**
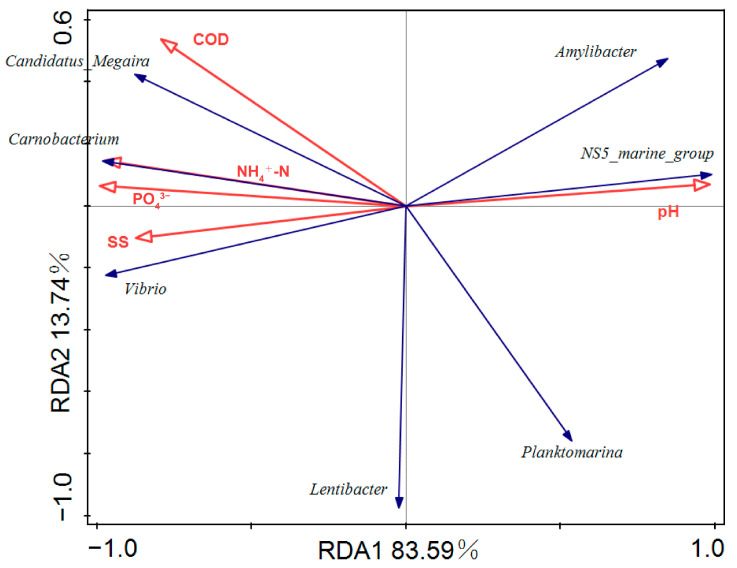
Redundancy Analysis (RDA) plot of the top three dominant genera in relation to environmental factors.

The top three dominant genera in the ABA, TA, and NBA, along with the main environmental factors, were selected for Redundancy Analysis (RDA) in this study in order to investigate the correlation between environmental factors and genus-level bacteria. The dominant genera included Vibrio, Carnobacterium, Candidatus_Megaira, Planktomarina, Lentibacter, Amylibacter, and NS5_marine_group. The environmental factors included pH, PO_4_^3−^, COD, NH_4_^+^-N, and SS. The RDA results showed that Vibrio, Carnobacterium, and Candidatus_Megaira were positively correlated with SS, NH_4_^+^-N, PO_4_^3−^, and COD and negatively correlated with pH. Vibrio exhibited a strong correlation with SS, PO_4_^3−^, and NH_4_^+^-N (Figure 6).

## 4. Discussion

### 4.1. Changes in Environmental Factors at Different Sampling Sites and RDA with Dominant Genera

The occurrence, development, and extinction of HABs are influenced by both abiotic and biotic factors. For instance, temperature, salinity, and nutrient concentrations are known to influence the occurrence of HABs. The change in environmental factors has an important effect on the occurrence of *N. scintillans* red tide. As a euryhaline eurytherm species, *N. scintillans* has a wide range of adaptations to temperature and salinity. Its suitable growth temperature is generally in the range of 10–28 °C, and the suitable salinity is generally in the range of 28–36 [22,23,24]. Nutrient salts are also important factors in controlling the growth of *N. scintillans* [25].

In this study, the NH_4_^+^-N, COD, PO_4_^3−^, and SS of the ABA were significantly higher than those of the other two sea areas and beyond the normal range. Studies have shown that red *N. scintillans* feeds on microorganisms such as microalgae, ciliates, and zooplankton eggs, releasing ammonia, phosphate, and other inorganic and organic substrates back into the surrounding waters [26,27,28]. Consequently, red tides caused by *N. scintillans* are often associated with elevated concentrations of ammonium and phosphate in the water [29]. Although the concentration of nutrients such as nitrogen and phosphorus did not directly affect the growth and reproduction of *N. scintillans*, the increase in its concentration could increase the abundance of phytoplankton and have a delayed and indirect effect on the growth and reproduction of *N. scintillans* by providing rich food [30].

The structure of microbial communities in aquatic environments is directly influenced by environmental factors [31]. Through a Redundancy Analysis (RDA) of dominant genera and major environmental factors, we identified SS, PO_4_^3−^, NH_4_^+^-N, and COD as the primary factors influencing *Vibrio* content. Wong et al.’s study [32] reveals that about 85% of *Vibrio* in tropical marine coastal waters reside in an attached state and correlate positively with the concentration of Total Suspended Solids (TSS). TSS is composed of various particulates, including chitin, planktonic organisms, cell debris, fecal matter, and resuspended sediments, all of which support the attachment of *Vibrio* spp. [33,34,35].

### 4.2. Differences of Bacterial Communities in Different Marine Areas

The content and community structure of marine bacteria are closely associated with the growth, composition, and physiological state of red tide algae. In this study, the Chao1 index was employed to assess the richness of bacterial species, where a larger index value indicates a higher species richness. The Shannon index and Simpson index were utilized to evaluate the diversity of microbial communities, with a higher index value signifying a greater species diversity. Comparing the three sampling areas, the Chao1 index, Shannon index, and Simpson index of the ABA were the lowest, indicating that the amount of *N. scintillans* in the ABA increased, and the richness, diversity, and evenness of the bacterial community were lower than those of the TA and NBA. Xia et al. [36] demonstrated that the bacterial community diversity reached the lowest value during the red tide extinction of *N. scintillans* in Hong Kong waters, which is in line with the lowest ABA diversity index in this study. The bacterial community diversity rose during the *Phaeocystis globosa* red tide outbreak and subsequently decreased as the red tide receded, a pattern that contrasts with the findings of this study [37]. The probable cause is that *Phaeocystis globosa* blooms provide a substantial organic matter input in the form of mucus, supporting very active microbial food webs from the onset to the end of the bloom [38].

In this study, the bacterial communities of the ABA, TA, and NBA of *N. scintillans* red tide were compared using high-throughput analysis. At the phylum level, Proteobacteria, Bacteroidetes, Firmicutes, Cyanobacteria, and Actinobacteria dominate the three sampling areas. Proteobacteria was the most dominant bacterium, and Bacteroidetes was the second-most dominant bacterium. At the genus level, *Vibrio* emerged as the primary dominant bacterium in both the ABA and TA. *Carnobacterium* constitutes the subdominant genus of the ABA. Prior research showed that the changes in bacterial community structure caused by different red tide species were different. *Phaeocystis globosa* is a toxin-producing red tide species that can produce hemolytic toxins, dimethyl sulfide (DMSP/DMS), thiopropionic acid, and other compounds to poison marine organisms [39]. Xu et al. [37] found that during a *Phaeocystis globosa* outbreak, Proteobacteria (51.09%) was the dominant microphylum in the surrounding sea area. The dominant genera were the *OM60 clade* (belonging to Proteobacteria), *NS5 marine group* (belonging to Bacteroidetes), and *Prochlorococcus*. Miguel et al. [40] found that in *Prorocentrum* red tide, Alphaproteobacteria, Gammaproteobacteria, and Bacteroidia were consistently dominant. Twelve taxa were defined as core members of the bacterial assemblage, representing the genera *Algiphilus*, *Cohaesibacter*, *Labrenzia*, *Mameliella*, *Marinobacter*, *Marivita*, *Massilia*, *Muricauda*, *Roseitalea*, and a few unclassified members of the Rhodobacteraceae. The core members are inferred to significantly contribute to primary and secondary metabolic functions but are not directly related to the virulence of dinoflagellates. Basu et al. [6] found that the amount of culturable bacteria during blooms was 2–3 times higher than that in non-bloom waters in the study of green *N. scintillans* red tide. The active phase flora was dominated by Gram-positive forms, a majority of which belonged to *Bacillus* of Firmicutes. As the bloom declined, Gram-negative forms emerged as dominant, and these belonged to a diverse γ-proteobacterial population consisting of *Shewanella* and equal fractions of a *Cobetia*–*Pseudomonas*–*Psychrobacter*–*Halomonas* population. There are some differences between the flora in red tide seawater and that of red *N. scintillans*, which may be related to the trophic mode of green *N. scintillans*.

Widely distributed in freshwater, coastal oceans, open oceans, and deep oceans, *Vibrio* constitutes an essential component of the microbiota of plants and animals in these aquatic environments and plays a crucial role in biogeochemical cycles and ecosystem health [41,42,43,44]. Some marine *Vibrio* bacteria are pathogenic and can not only inflict harm on the aquaculture industry but also pose a threat to human health via the food chain [45,46,47]. These include *Vibrio cholerae*, *Vibrio vulnificus*, *Vibrio Parahaemolyticus*, and *Vibrio alginolyticus*. A study of the red tide of *N. scintillans* in Clear Water Bay, Hong Kong, revealed that the relative abundance of the *Vibrio* family bacteria underwent a sharp increase, emerging as the most abundant bacteria in the *N. scintillans*-related bacterial community (ranging from 73.8% to 78.3% of the total community) [48]. The content of *Vibrio anguillarum* was the highest. This is a pathogenic bacterium that causes diseases in marine fish, and the hemorrhagic septicemia caused by this bacterium is regarded as a major disease in marine fish, resulting in substantial economic losses [49].

*V. splendidus* is the most dominant species in this study, accounting for 93% of the total *Vibrio*. This species is an important opportunistic pathogen in aquaculture in marine environments. It can cause a variety of host diseases in organisms such as echinoderms [50], bivalve shellfish [51,52], and fish [53,54], resulting in incalculable losses in the aquaculture industry. It was found that *V. splendidus* causes disease mainly through the production of virulence factors that lead to disruption of metabolism and biological functions of the organism [55,56]. Its virulence factors mainly include adhesion factors, extracellular products, lipopolysaccharides, hemolysins, and metalloproteinases [57,58].

The genus *Carnobacterium* consists of inactive lactic acid bacteria that are considered to be bioprotective bacteria in seafood but have since been found to be the main spoilage bacteria in frozen vacuum packaged or modified atmospheric packaged seafood [59]. Recent studies have shown that *Carnobacterium* may be the major spoilage bacteria in seafood [60,61]. Calliauw et al. [62] found that *Carnobacterium* is a major spoilage bacterium in peeled brown shrimp stored at 4 °C for 7 days. Macé et al. [63] investigated the spoilage of cooked tropical whole shrimp and found that *Carnobacterium* can lead to the rapid deterioration of seafood and produce volatile organic compounds in the process. Certain strains of *Carnobacterium* are pathogenic to fish and can cause various pathological changes in different fish.

## 5. Conclusions

In this study, the concentrations of NH_4_^+^-N, COD, PO_4_^3−^, and SS in the ABA water were higher than those in the TA and NBA. The water quality of the ABA was inferior to that of the fourth category of seawater evaluation standards, indicating that the water environment deteriorated during the outbreak of *N. scintillans*. The richness, diversity, and uniformity of the ABA bacterial community declined. *Vibrio* was the most dominant genus in the ABA. Of the 15 *Vibrio* species identified in this sample, *V. splandidus* was the most dominant species in the ABA, accounting for 93% of *Vibrio*. *Carnobacterium* was the second-most dominant genus. In the ABA, the combined content of *Vibrio* and *Carnobacterium* exceeded 50%. *V. splandidus* is a pathogenic bacterium for shellfish and sea cucumber, and *Carnobacterium* is a common spoilage bacterium in seafood. This may be one of the reasons for the death of marine organisms caused by the red tide of *N. scintillans*.

## Figures and Tables

**Table 1:** Physicochemical indexes in different sampling areas.

Sample		ABA	TA	NBA
Temperature	°C	22.40 ± 0.02 ^c^	21.00 ± 0.17 ^b^	20.33 ± 0.07 ^a^
Salinity		32.20 ± 0.14 ^b^	31.20 ± 0.43 ^a^	31.14 ± 0.06 ^a^
pH		7.05 ± 0.07 ^a^	7.44 ± 0.05 ^b^	8.45 ± 0.03 ^c^
COD	mg/L	58.10 ± 0.12 ^c^	5.39 ± 0.00 ^b^	0.85 ± 0.01 ^a^
NH_4_^+^ N	mg/L	13.35 ± 0.15 ^c^	6.71 ± 0.03 ^b^	0.01 ± 0.00 ^a^
PO_4_^3−^	mg/L	1.26 ± 0.02 ^c^	0.75 ± 0.01 ^b^	0.01 ± 0.01 ^a^
SS	mg/L	180.00 ± 24.45 ^b^	145.00 ± 42.35 ^b^	46.00 ± 9.90 ^a^

Data represent the mean ± SD (standard deviation) of triplicate measurements. Different lowercase letters indicate statistically significant differences; the significance level is *p* < 0.05. Abbreviations for the sampling sites are as follows: ABA: Algal Bloom Area, TA: Transition Area, and NBA: Non-Algal Bloom Area.

**Table 2:** Diversity of bacterial communities in different sampling areas.

SampleID	Sequence Number	Chao1 Index	Shannon Index	Simpson Index	OTUs	Goods_Coverage
ABA	36835	656.79 ± 18.70 ^a^	4.41 ± 0.23 ^a^	0.88 ± 0.01 ^a^	497 ^a^	0.99
TA	59097	834.87 ± 22.84 ^c^	5.41 ± 0.14 ^b^	0.94 ± 0.01 ^b^	617 ^b^	0.99
NBA	53651	747.89 ± 51.76 ^b^	6.37 ± 0.03 ^c^	0.97 ± 0.00 ^c^	575 ^b^	0.99

Data represent the mean ± SD (standard deviation) of triplicate measurements. Different lowercase letters indicate statistically significant differences; the significance level is *p* < 0.05. Abbreviations for the sampling sites are as follows: ABA: Algal Bloom Area, TA: Transition Area, and NBA: Non-Algal Bloom Area.

**Table 3:** Abundance ratios of dominant genera in different noctilucent species.

Generic Name	ABA	TA	NBA
*g__Vibrio*	34.38	18.43	-
*g__Carnobacterium*	16.57	2.33	-
*g__unidentified*	8.25	4.80	19.28
*g__Candidatus_Megaira*	5.69	-	-
*g__Planktomarina*	5.04	10.88	9.53
*g__uncultured*	4.91	13.77	8.56
*g__uncultured_bacterium*	3.75	-	1.12
*g__Pseudoalteromonas*	3.48	2.90	-
*g__Glaciecola*	2.40	7.77	-
*g__Lentibacter*	1.99	8.31	2.07
*g__Amylibacter*	1.31	-	7.32
*g__NS5_marine_group*	-	-	6.93
*g__Aurantivirga*	-	1.21	5.40
*g__marine_metagenome*	-	-	4.36
*g__NS4_marine_group*	-	-	4.27
*g__Clade_Ia*	-	-	3.37
*g__Yoonia-Loktanella*	-	2.38	2.88
*g__Candidatus_Actinomarina*	-	-	1.87
*g__OM60_NOR5_clade*	-	-	1.64
*g__unidentified_marine_bacterioplankton*	-	-	1.55
*g__Salinisphaera*	-	-	1.45
*g__NS3a_marine_group*	-	1.89	-
*g__Jannaschia*	-	3.48	-
*g__Litoricola*	-	1.46	-
*g__Nautella*	-	1.83	-
*g__Marinomonas*	-	1.93	-

Abbreviations for the sampling sites are as follows: ABA: Algal Bloom Area, TA: Transition Area, and NBA: Non-Algal Bloom Area.

**Table 4:** Proportion of vibrio species in different algal environments.

Specific Name	ABA	TA	NBA	Mean Value
*Vibrio_splendidus*	0.97	0.97	0.86	0.93
*Vibrio_harveyi_NBRC_15634_ATCC_14126*	0.01	0.01	0.04	0.02
*uncultured_Vibrio_sp.*	0.01	-	0.01	0.01
*Vibrio_cyclitrophicus_FF75*	-	-	0.02	0.01
*Vibrio_halioticoli_NBRC_102217*	-	-	0.02	0.01
*Vibrio_sp._INTCD148H*	-	-	0.02	0.01
*Vibrio_sp._H075*	-	-	0.01	-
*Vibrio_cholerae*	-	-	0.03	0.01

Abbreviations for the sampling sites are as follows: ABA: Algal Bloom Area, TA: Transition Area, and NBA: Non-Algal Bloom Area.

## Data Availability

All data are included in the paper.
